# Personal Explanations for Psychosis: A Systematic Review and Thematic Synthesis

**DOI:** 10.1093/schizbullopen/sgaf006

**Published:** 2025-03-04

**Authors:** Benjamin-Rose Ingall, Merly McPhilbin, Felix Lewandowski, Yasuhiro Kotera, Gerald Jordan, Mike Slade, Fiona Ng

**Affiliations:** School of Health Sciences, Institute of Mental Health, University of Nottingham, Nottingham NG7 2TU, United Kingdom; School of Health Sciences, Institute of Mental Health, University of Nottingham, Nottingham NG7 2TU, United Kingdom; School of Psychology, University of Nottingham, Nottingham NG7 2TU, United Kingdom; School of Health Sciences, Institute of Mental Health, University of Nottingham, Nottingham NG7 2TU, United Kingdom; Centre for Infectious Disease Education and Research, Osaka University, Suita, Osaka 565-0871, Japan; School of Psychology, University of Birmingham, Birmingham B15 2TT, United Kingdom; School of Health Sciences, Institute of Mental Health, University of Nottingham, Nottingham NG7 2TU, United Kingdom; Health and Community Participation Division, Faculty of Nursing and Health Sciences, Nord University, 7801 Namsos, Norway; School of Health Sciences, Institute of Mental Health, University of Nottingham, Nottingham NG7 2TU, United Kingdom

**Keywords:** meaning making, sense making, personal explanations, psychosis, schizophrenia, systematic review

## Abstract

**Background and Hypothesis:**

Psychosis refers to the state whereby one’s experience of reality differs from those around them. The ineffability of psychosis does not render the experience void of meaning, and the ways individuals integrate their experiences of psychosis into their life narratives cannot be dismissed. Meaning is an essential part of recovery. This review aimed to identify categories of personal explanations that people with psychosis use to explain their experiences.

**Study Design:**

This systematic review is based on a preregistered protocol (CRD42023421125). Four databases, MEDLINE, Embase, Scopus, and PsycINFO, and 5 journals were searched April to November 2023. Qualitative and mixed-methods studies that explored the personal explanations employed by adults who experience psychosis, regardless of diagnostic status, were included.

**Study Results:**

Twenty-five studies met the inclusion criteria, representing the views of 682 participants from 15 countries. Included studies were appraised using the CASP Qualitative Studies Checklist.

**Results:**

were synthesized using thematic analysis. Personal explanations for psychosis experiences were grouped into 5 themes: Physical and psychiatric; Traumatic and adversarial; Emotional; Religious, spiritual, and magical; No explanation. Participants reported multiple explanations for their experiences.

**Conclusions:**

Individuals with experience of psychosis seek to explain these experiences, and these personal explanations may be multiple and complex in nature. The identified personal explanations can be used to further explore the ways that people situate their experiences into their personal context. This understanding should be utilized by professionals to support the provision of recovery-oriented care, with implications for assessment, treatment, intervention, and recovery outcomes.

## Background

There is continuing debate regarding the nature and classification of mental health. A recent quasi-systematic review of models of mental health identified 34 different models across 5 categories, including biology, psychology, social, consumer, and cultural.^1^ Biological and psychological models were more frequently endorsed compared to social, consumer, and cultural models. Specifically for psychosis, the value of current categorical classification (which is based on biological and psychological models) has been questioned. For example, in a global study of 701 individuals who take antipsychotic medication found that people are 13 times more likely to attribute experiences to solely social causes, rather than biological or genetic causes.^2^

There is a growing evidence base for understanding psychosis on a continuum.^3^ Conceptualizing psychosis as on a continuum would account for similar experiences in populations who are considered nonclinical.^4^ Indeed, such experiences do not appear to differ between those with and without a need for care.^5^ Anomalous experiences are common and not limited to clinical populations, where 75% of British adults reported having ever had an anomalous experience (e.g., out-of-body experiences/seeing ghosts), and 48% reported having them “sometimes” or “often.”^6^ Psychiatric nosology utilizes symptomatic classification rather than etiological, as for many diagnoses, the underlying cause is either not known or not understood. In practice, clinicians judge the etiology of mental disorders to be on a spectrum from psychosocial to biological, with psychosis-spectrum conditions falling on the biological end of this spectrum.^7^ A shift towards a continuum or dimensional understanding of mental health problems has emerged. One example of a dimensional model is mechanistic property clusters, which suggest that psychiatric conditions can be classified as clusters of experiences based on shared biological causes.^8^ A second example of a dimensional approach is the Hierarchical Taxonomy of Psychopathology (HiTOP) model organizes that mental health problems and its subtypes into observed symptoms, where similar symptoms are grouped together to reduce heterogeneity and identify comorbidities.^9^

The ineffability of psychosis does not render the experience void of meaning. Psychosis is described as having a profound impact on one’s sense of meaning and of identity, and individuals face the task of rebuilding their identity and meaning in life.^10^ Identity can be considered intrinsically linked to the integration of events into the life narrative, with narrative identity consisting of an ever-evolving story that one utilizes to make sense and meaning of their lives and selfhood,^11^ and is especially important in the context of challenging experiences.^12^ Psychosis experiences have historically been rendered as meaningless indicators of dysfunction.^13^ People with psychosis have been considered to lack insight into their condition if they did not consider themselves ill.^14^ Lacking insight into illness, or *anosognosia*, is considered a common feature of psychosis, according to the DSM-V.^15^ The concept of clinical insight is defined in a number of ways but includes the acceptance of illness, the limitations it causes, and the need for treatment.^16^ Clinical insight fails to account for the numerous levels of ways people explain their experiences and can be considered to be a Western, biologically reductionist position.^17^ To dismiss the lived experience narratives as invalid is a form of epistemic injustice. The narrative insight model considers insight to be a personally constructed narrative that explains ones experiences in the context of their life as a whole and which can be understood by others.^18^ This corresponds with the model of narrative integration, whereby an experience that conflicts with the fundamental human need for understanding, such as psychosis, requires a degree of narrative meaning-making in order to integrate it into the life narrative as a whole.^19^ A core feature of the experience of psychosis is the disruption of the self-world relationship leading to a change of identity, contingency, and coherency. As such, a key part of recovery is the process of integrating the experiences into ones’ changed identity and meaning in life in order to reestablish a sense of coherency in the life narrative.^20^ Individuals develop a personal explanation for the experience to integrate it into their life narrative.^21^ As such, meaning-making and the ways individuals integrate their experiences of psychosis into their life narratives cannot be dismissed.

Meaning-making has been identified as playing an important role in managing the impact of psychosis,^22^ and research suggests that it maintains relevance throughout life, from the first episode of psychosis^23^ to decades after diagnosis.^24^ Meaning-making, including the development of a personal explanation, has been found to play a pivotal role in consolidating ones sense of self and in experiences of recovery after a first episode of psychosis.^25^ Indeed, for many, the rebuilding of a sense of continuity through the development of meaningful understanding of their psychosis experiences was, itself, recovery.^20^ Studies examining meaning-making in psychosis have predominately been qualitative in nature, particularly utilizing a life story approach. One study interviewed 20 individuals with psychosis from Finland identified that a minority of individuals reported their experiences of psychosis as a crisis that disrupted their life course and as an expected reaction to adversity.^24^ This was similarly reported in a Dutch qualitative study where individual narratives revolved around the exposure to trauma affecting life, difficulties accessing treatment, with disclosure reducing a sense of alienation.^26^ Set against the backdrop of taxonomic uncertainty and the clear narrative of the importance of meaning from people with lived experience, there is immense value in the consideration of the personal explanations people use to explain their experiences.

Despite increased interest into meaning-making in psychosis, there is no existing framework of personal explanations that people use to explain their psychosis. Presently, research into personal explanations use a variety of different categories to describe their findings, eg, esoteric factors,^27^ cultural factors,^28^ spiritual/mystical factors,^29^ highlighting the need for a comprehensive framework. This review aimed to provide a comprehensive and in-depth understanding of the personal explanations people use to explain their experiences of psychosis. The objectives were (1) to develop an understanding of the personal explanations people attribute to their experiences of psychosis and (2) to explore differences in personal explanations between subgroups.

## Methods

A qualitative systematic review was conducted following the ENTREQ (enhancing transparency in reporting the synthesis of qualitative research) statement^30^ and PRISMA guidelines.^31^ The protocol was registered with PROSPERO in April 2023 (CRD42023421125).

### Information Sources

Papers were sought through 5 routes: (1) Electronic databases (*n* = 4) were searched from April to November 2023; MEDLINE ALL (Ovid), Embase (Ovid), Scopus (Elsevier), and PsycINFO (ProQuest); (2) Hand searching journal table of contents (*n* = 5): “Psychosis,” “Schizophrenia Bulletin,” “Qualitative Health Research,” “Journal of Mental Health,” and the journal which yielded the highest number of eligible papers from the database search; (3) Google Scholar search engine; (4) Experts in the field were consulted to identify further relevant publications; and (5) forward and backward citation searching of all included publications.

#### Inclusion Criteria

Studies were eligible for inclusion if they (1) were empirical, (2) utilized a qualitative design, or mixed-method where the qualitative aspect was extractable, (3) consisted of adults (18+ years) with psychosis or psychosis-like experiences (diagnosed or self-reported), (4) included the personal explanations participants attributed to their experiences, (5) were written in the English language. Psychosis-like experiences with an organic cause, such as dementia, were excluded. There was no restriction on publication year. We used a broad definition of personal explanations to maximize inclusion and diversity of perspectives in the review.

#### Electronic *Search Strategy.*

The search strategy was developed and undertaken in collaboration with a senior information specialist. The qualitative search strategy tool SPIDER (sample, phenomena of interest, design, evaluation, research type)^32^ was utilized to define key areas of the search terms. Keywords included “psychotic disorder,” “spiritual emergency,” “explanatory model.” Google Scholar search terms included “psychosis” OR “delusion” OR “hallucination” AND “cause” OR “explanation” AND “interview” (see Supplementary Material 1 for full search strategy).

#### Procedures

**Figure 1.**
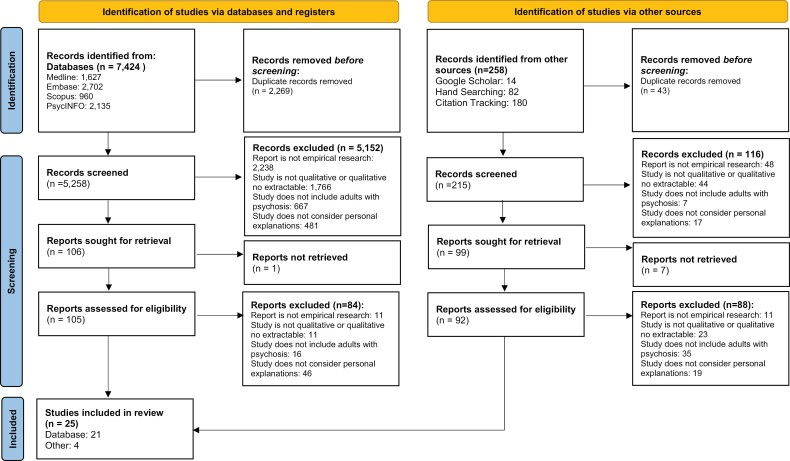
PRISMA Flowchart of Included Studies

Identified citations were exported to Endnote where duplicates were removed and exported to Rayyan software for screening. Title and abstract were screened against inclusion criteria by BRI, FL, and YK, where consensus was reached through discussion. Citations meeting the inclusion criteria were extracted for full-text screening. See Supplementary Material 2 for full screening criteria for inclusion. The core reviewing team consisted of individuals with expertise in psychology, mental health recovery, neurodiversity, and experience as a person with LGBTQ + identity. After removal of duplications, the title and abstract of 5473 papers were screened against the inclusion criteria, with 5268 removed and 205 were sought for a full-text screening. Of those sought, 8 were unable to be retrieved, 172 were excluded on a full-text screening against the inclusion criteria, and 25 were included in the review (Figure 1).

#### Quality Appraisal

Included studies were quality appraised by BRI and MM using the CASP Qualitative Studies Checklist tool. Any areas of discordance were discussed until consensus was achieved. A numerical value was assigned to questions, with a lower score indicating lower quality.

#### Data Analysis

All text in findings or results sections of included papers was extracted to the software NVIVO12. A thematic synthesis was conducted. Analysis consisted of the 3 stages described by Thomas and Harden.^33^ First, papers were coded line-by-line to identify relevant findings. Second, similar codes were grouped into descriptive themes. Third, analytic themes were developed through interpretation of the descriptive themes, based upon the research question. For each paper, all text relating to findings was coded by BRI to develop the initial codebook. MM independently coded 25% to refine the codebook, and then an additional 25% for concordance. A coding diary was kept throughout the process. This was used to note decision-making, coding changes, and justifications. A subgroup analysis was conducted utilizing the CASP score for each paper. A second subgroup analysis was conducted using the Healthcare Access and Quality (HAQ)^34^ index as a guideline to categorize papers according to their country of study. The HAQ is a standardized, global measure of the accessibility and quality of healthcare, on a scale of 0-100, with 100 being the highest quality, most accessible healthcare.

### Findings

#### Study Characteristics

Twenty-five studies were included in the review.^24,27–29,35–55^ Studies were conducted across 15 countries, most frequently England. All studies were published between 1998 and 2023. For the included studies, data regarding study aim, location, recruitment strategy, participants, and methodology, and analysis were extracted and are presented in Supplementary Material 3.

Included papers reported findings from a total of 682 individuals with experiences of psychosis. Participants were primarily male (*n* = 420; 62%), and 2 papers were conducted with an exclusively male sample. Ethnicity was reported for 35% of participants, most frequently White British (*n* = 88). Diagnosis was reported for 66% of participants; most frequently schizophrenia (*n* = 340; 50%). No paper identified participants as without a diagnosis. Healthcare use was reported for 91% of participants, with 87% (*n* = 593) currently utilizing mental health services or medication.

### Objective 1: Personal Explanations of Psychosis

Seven themes of personal explanations were identified: Medical model explanations; Drug-related explanations; Physical stress explanations; Traumatic and adversarial explanations; Emotional explanations; Religious, spiritual, and magical explanations; No explanation (Table 1). Themes are not hierarchal and have been ordered according to their proximity to the medical model. The full codebook with illustrative quotes can be found in Supplementary Material 4. Of the 25 papers, 15 noted that participants held multiple personal explanations for their experiences simultaneously. For some, these were conflicting, but for others, these were held in tandem, such as an interpersonal conflict leading to a curse (see Supplementary Material 4 for reference key).

**Table 1.** Themes and Subthemes

Theme	Subthemes	Number of papers
Medical model explanations		19
	Physical Illness or Injury	13
	Genetics	8
	Psychological Illness	7
Drug-related explanations		9
Physical Stress		4
Trauma and adversities as explanations		16
	Chronic or complex trauma	10
	Single event trauma	8
	Unspecified trauma	7
	Adversity	4
Emotional explanations		20
	Interpersonal difficulties	15
	Emotional experiences	15
	Transitional periods	3
Religious, spiritual, and magical explanations		22
	Religious entities and phenomena	14
	Spiritual entities and phenomena	14
	Magical entities and phenomena	11
No explanation		9
	Explanation not known	6
	Explanation not sought	2
	Explanation not found	2

#### Medical *Model Explana*tions

The medical model refers to biomedical explanations of mental illness, which is often used by health professionals to diagnose and treat illness. Whilst it is the dominant model in discourse, it minimally considers the role of the psychological and social contributors to health. Medical model explanations were provided in 19 papers, which included biological causes and injury,^27–29,37,38,40,41,43–45,47,52,54^ genetics,^27,29,39,41–43,45,53^ and psychological illness.^35,39,40,45,52,53,55^ Some participants explained their experiences of psychosis as having a physical cause, such as disease, chemical imbalance, brain dysfunction or injury, or resulting from another physical ailment. Others explained their experiences as resulting from psychological illness, either a psychotic disorder or as resulting from unmanaged mood disorders or as a genetic phenomena, or as resulting from genetic inheritance.

*“I believe that they come from inside my brain… Some brain dysfunction.”* ^36^

*“But I think there’s definitely something there genetically. It runs in the family because [my mum’s] mum was diagnosed… Paranoid psychosis.”* ^53^

*“I feel like they all linked, like the psychosis came along with a lot of depression, anxiety.”* ^35^

#### Drug-Related Explanations

The impact of drugs^27,39,41,43–46,50,52^ was identified as a primary or contributory role in their experiences of psychosis in 9 papers, with alcohol, prescription, and nonprescription drugs commonly discussed.

*“I think it [narcotics] was definitely a contributing factor but I don’t think it was the sole reason why I lost my marbles.”* ^41^

#### Physical Stress Explanations

Four papers discussed the experiences of psychosis as resulting from physical stress,^46–49,52^ such as overwork, or not having met their needs, such as food and sleep.

*“I wasn’t sleeping, it must have been, and starving myself, must have brought it on.”* ^48^

#### Trauma and *Adversities* as *Explanations.*

Sixteen papers discussed experiences of psychosis as a consequence of trauma and adversity, including chronic or complex trauma,^24,37,38,41,43,46,48,49,52,55^ single event trauma,^24,29,36,37,41,44,47,55^ unspecified trauma,^27,36,38,39,41,49,52^ and adversity.^27,37,44,47^

Chronic and complex trauma (10 papers) referred to multiple, long-lasting, repeated, or continuous trauma and included abuse and neglect, warfare, political violence, and imprisonment, exile, seeking refuge, and family separation.

*“I was, like, abused and all that when I was younger. But I don’t want to use that as an excuse, but it’s still in my mind and stuff, and I know that it shattered my personality as a young kid, and I’ve never been able to repair that.”* ^49^

Single-event trauma (8 papers) referred to a traumatic event that impacted an individual one time. This theme included bereavement, assault, and accident, or injury.

*“It has something to do with the bereavement I imagine, alright… I didn’t take time to grieve, I just wanted to get the thing settled… It was only with the dying off of the others that I had this terrible coping.”* ^47^

Unspecified trauma (7 papers) referred to traumatic events which were considered to explain psychosis, but which were not able to be specified as single event or chronic/complex.

*“[Voices are] from a traumatic episode where you’re just becoming your own best friend… I believe it all started off as a traumatic experience.”* ^39^

Four papers discussed the effects of adversity through pervasive, societal hardships as an explanation for experiences. This consisted of poverty, homelessness, debt, discrimination, social exclusion, and societal and political structures.

*“People should have money and should be given jobs because if you do not have money, you become stress[ed]. This stress exacerbates your illness. When you have money you don’t become mentally ill.”* ^44^

#### Emotional *Explanations.*

Twenty papers discussed emotional explanations consisting of emotional experiences,^24,27–29,35,39,41–43,47,49–52,55^ interpersonal difficulties,^24,27–29,35,36,42–45,47,50–52,54^ and transitional life periods.^24,46,50^

Personal explanations of psychosis, which encompassed emotional experiences (15 papers) included emotional stress, emotional suppression, difficulty with emotional insight, psychosis as a problem of the nerves, and as resulting from being sensitive by nature.

*“I worked for the post office for many years and I was good at it... and then I got fired. Since then, I haven’t been able to find another job... I got depressed, so I decided to go to Israel... and here, in Israel, I became really ill with schizophrenia.”* ^42^

Fifteen papers discussed explanations resulting from interpersonal difficulties, including social altercations, rejection, regrets, isolation and loneliness, social anxiety, and relationship conflicts and breakdowns.

*“There were constant disappointments in our relationship and then that divorce thing happened. It affected my self-confidence, leading to other failures and disappointments. It was as if the house of cards that we had carefully built suddenly collapsed.”* ^24^

Transitional life periods (3 papers) referred to a period of change, including transitioning to adulthood, to married life, and to motherhood.

*“It was OK until, as I say, I went to senior school, and then things got difficult, and you don’t really talk about problems and things like that cos nobody really understands about it. It all just got bottled up and bottled up and I reckon that is what really caused it, well one of the reasons.”* ^50^

#### Religious, *Spiritual*, and *Magical Explanations.*

Religious, spiritual, and magical explanations for psychosis experiences were discussed in 22 papers. Three subthemes emerged: religious entities and phenomena,^29,37–42,46–49,51,53,54^ spiritual entities and phenomena,^27,29,35,36,38,40,46,48,50–55^ and magical entities and phenomena.^27,29,35,37,42–44,47,51,52,54^ Religious entities and phenomena were discussed in 14 papers and examples of themes included the will of Allah or God’s plan, punishment from God, the presence of God or the Holy Spirit, and the role of a higher power generally.

*“Of course this is because of God. I mean, God gives you these problems but he also gives remedy.”* ^48^

Spiritual entities and phenomena referred to in 14 papers and involved the role of something greater than the self, but which was not strictly related to religion. Some papers explained experiences through spiritual frameworks prevalent in their culture, such as the role of djinn (spirits), while others explained their experiences in more generalized terms, emphasizing the experience as a spiritual connection, but without specifying the nature of said connection. The emotional valence of these explanations varied; for some, the experience was considered a gift, and for others, a burden.

*“Djinns have come from time to time, they talk to me, and I hear what they tell me…”* ^36^

*“There is the spiritual part there. There is a definite spiritual connection there.”* ^40^

Explanations from a magical origin (11 papers) were described as negative explanations, and examples included curses, witchcraft, and black magic. The negative impact was particularly reported through difficulties with discussing these explanations with healthcare professionals.

*“There are bad people and witchcraft do exist and that was what happened to me. I did not believe in witchcraft before that, but now I do.”* ^37^

*“I’ll say “I hear a presence that’s a divinity…” And my… medical person is sitting there going ‘crazy box.’ There’s a filter that rejects that experience.”* ^39^

#### No Explanation

Some participants discussed not knowing why they experienced psychosis. This theme consisted of not knowing,^27,29,35,51,52,54^ not seeking,^41,49^ and not finding an explanation.^41,55^

It was unclear in some papers (6 papers) whether participants had sought an explanation or not for their psychosis experiences. Two papers discussed participants had not sought an explanation for their psychosis, frequently not wishing to dwell on the experiences. Whilst, in 2 papers, participants discussed seeking an explanation but had not developed a personal explanation yet.

*“I dunno. It’s just, there’s something unexplainable, to be honest.”* ^*44*^

*“I have not really put thought into it because it’ll just mess my head up if I put thought into it.”* ^41^

*“There has got to be a reason why, that’s what I wanted to find out, if you get a cut on your hand you can see it, you can see it getting worse, whereas something inside you can’t see. There is nothing obvious.”* ^41^

### Objective 2: Subgroup Analysis

#### CASP Quality Analysis

A common feature of low-scoring papers was inadequate consideration of the participants–researcher relationship and of ethical issues. When studies scoring 21 or lower on the CASP (n = 4) were removed from the analysis, there was no change in the pattern of thematic endorsement (Table 2).

**Table 2.** CASP Quality Subgroup Analysis

	All papers *N* = 25		Excluding low CASP *N* = 21	
	*n*	%	*n*	%
Physical, psychiatric	21	84%	19	90%
Trauma, adversities	16	64%	14	67%
Emotional	20	80%	17	81%
Religious, spiritual, magical	22	88%	18	86%
Not known	9	36%	6	29%

#### Healthcare Access and Quality

**Figure 2.**
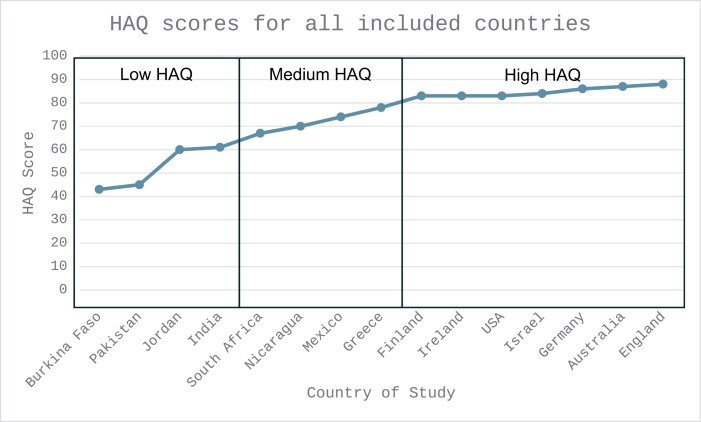
HAQ Scores by Country of Study

HAQ scores for all countries of the studies were retrieved (Supplementary Material 5). These ranged from 43 to 88 and were segmented into 3 groups using a line graph: scores 61 or lower were “low,” between 62 and 82 were “medium,” and 83 and above were “high” (Figure 2). Within each study, participants endorsed multiple themes. For the purposes of the subgroup analysis, the dominant theme presented in each study was utilized.

**Figure 3.**
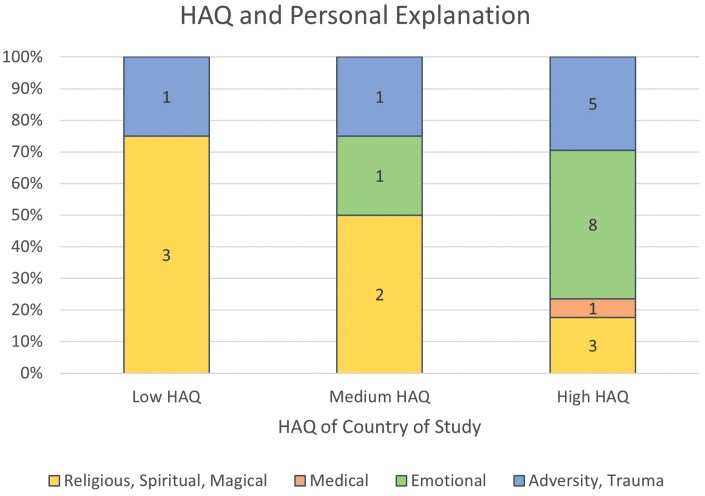
HAQ and Personal Explanation

Participants in countries with a low HAQ primarily held personal explanations related to religion, spirituality, magic, and trauma and adversity. Participants in countries with a medium HAQ endorsed explanations related to religion, spirituality, and magic; emotions; and trauma and adversity. Finally, participants in countries with a high HAQ endorsed all 4 themes. It appeared that emotional explanations were more highly endorsed in countries with a higher HAQ, while participants in countries with a lower HAQ favored religious, spiritual, and magical explanations (Figure 3).

## Discussion

Whilst there are other systematic reviews published on personal explanations in psychosis,^56,57^ this review contributes a unique framework of personal explanations people hold in response to experiences of psychosis. Explanations include; Physical and psychiatric explanations; Traumatic and adversarial explanations; Emotional explanations; Religious, spiritual, and magical explanations. Religious, spiritual, and magical explanations are commonly less reported in depth; this paper extends this knowledge by thematically classifying these explanations.

In this review, the most common forms of explanation for psychosis were religious, spiritual, magical, emotional, and medical model-related. The almost equal endorsement of these explanations highlights the need for increased understanding of alternative explanations within people with lived experience, society, and clinical practice. This may be attributable to the identification of a larger proportion of papers in this review from the Global South compared with the scoping review. The current findings are in line with findings from a scoping review of causal beliefs of psychosis amongst health professionals and people with lived experience, where 6 causal beliefs, including biogenetic, psychosocial, spiritual/religious, substance-related, a part of personal characteristics, and a part of the human experience, were identified.^58^

Papers frequently discussed participants having more than one explanation for their experiences. A previous review on explanatory models and psychosis outcome noted that complex or multiple explanations were common and could be considered related or unconnected.^59^ This is reported as the norm in non-Western cultures and is observed in Western cultures as well.^60^ Multiple explanations may be considered the norm in non-Western cultures due to the apparent incompatibility of religious, spiritual, and magical explanations with the medical model. For example, to a Western observer, the explanation of Djinn possession may appear incompatible with a medical understanding of mental illness, due to a lack of cultural conception of Djinn. However, an explanation of a traumatic experience leading to psychosis might be considered more congruent with a medical understanding due to biopsychosocial models. It is important to note that explanations which are considered contradictory by an outside perspective may be coherent from the individuals frame of reference, as well as to those with whom they share cultural references.

The identification of religious, spiritual, and magical beliefs in this review highlights the importance of belief alliance between health professionals and people with psychosis. In this review, some participants felt that their religious, spiritual, and magical beliefs were not or would not be respected by mental health professionals. Empirically, discrepancies in causal beliefs between professionals and people with psychosis have been reported in the literature, with professionals more likely to endorse biogenetic factors and people with psychosis more likely to endorse social and societal factors.^58^ For example, a systematic review of spirituality among people with mental health difficulties found that their experiences of spirituality were similarly dismissed, misunderstood, or pathologized in healthcare.^61^ However, accounting for an individual’s spiritual needs in healthcare is associated with a stronger therapeutic relationship.^62^ Clinicians may be wary of discussing religion, spirituality, and magic in the context of psychosis and may pathologize such explanations. However, a religious, spiritual, or magical explanation that is personally constructed and socially coherent to explain an experience of psychosis within one’s life narrative should not be considered indicative of pathology. Coherency to others, especially with reference to religious, spiritual, and magical explanations, is embedded in one’s cultural context. Sensitive consideration of an individual’s beliefs is an important aspect of culturally competent healthcare, strengthening the therapeutic relationship and promoting shared decision-making. Additionally, one included paper^27^ described religious, spiritual, and magical explanations as causing helplessness and anxiety due to an external locus of control. However, this was not always the case, with participants utilizing specific methods of symptom management that aligned with their personal explanation. It may therefore be important to consider in more depth the ways in which people explain their experiences through religious, spiritual, and magical means. In other literature on health and meaning-making, the concepts of ittikal (dependence) and tawakkul (reliance) on God are differentiated.^63^ Understanding illness as the will of God did not prevent people from considering it their responsibility to exert their agency in seeking treatment. Religious, spiritual, and magical explanations are not automatically incompatible with a sense of agency and control, nor with seeking medical intervention.

Individual’s personal explanation(s) for their experiences should be considered with sensitivity and respect. This information can be utilized within individual support plans, supporting an individual to feel heard and respected, and forms of support, symptom management, and/or treatment that align to their personal explanations can be explored. The suggested framework can be used as basis for considering personal explanations of psychosis within healthcare through providing initial prompts for exploration.

### Implications and Future Research

That individuals commonly utilized nonclinical frameworks to explain their experiences of psychosis has implications for practice. It is important that an individual’s personal explanations are sensitively considered by any professional to facilitate a beneficial therapeutic relationship and to embed personal understandings within any treatment plans. However, it is also to ascertain whether individuals with psychosis want to make meaning from their experiences. The review identified that not seeking an explanation was present in some studies, indicating that meaning-making should not be forced upon individuals.

Given the qualitative differences between clinical and personal explanations, innovations in the assessment of insight (such as the Mental State Examination) may benefit from the inclusion of personal explanations. Whilst there is a need to balance the clinical and personal perspectives, the inclusion of personal explanations nuance the impact of psychosis. Integration of meaning-making processes in routine care for psychosis may support people to develop their own explanations for their experiences. Further training for clinicians may be required to reduce the potential perceived stigma associated holding alternative explanations.

Future research can focus on individual perspectives for support, intervention, and treatment preferences with regard to their personal explanations. Emphasis on the spiritual, religious, and magical explanations could be a starting point. Consideration over the duration needed, the mechanisms and processes by which meaning-making in psychosis occurs may support the development of new interventions or in the integration of meaning-making processes into usual care.

### Limitations

There are 5 main limitations associated with this review. First, participants in the included studies were predominately recruited through mental health services, with limited reporting of participant–clinician relationship. This may have had an impact on participant disclosure and may have primed certain responses, as participants may be less likely to speak freely.

Second, despite our intentions to explore differences across cultures, there was a disproportionate number of included studies that were based in England. Whilst this may have been overcome by having a less restrictive exclusion criteria, it may indicate either there are more empirical studies conducted on meaning-making in WEIRD (Western, Educated, Industrialized, Rich, Democratic) settings or that the search strategy and research team could have benefited from wider cultural input.

Third, understanding the perspectives of people from countries other than England (particularly non-Western contexts) may be more challenging when only including empirical studies published in English. The interviews of a minority of included studies were conducted in languages other than English. However, differences between studies in interpretation and translation methods may have increased the risk of translation bias. Inclusion of languages other than English may have provided greater understanding and enhanced the inclusivity and diversity of included papers in this review. This is particularly pertinent given the finding that people explain psychosis using religious, spiritual, and magical explanations. Additionally, conducted targeted literature searches specific to non-Western contexts may have bolstered the number of included papers beyond the Western context.

Fourth, the sole use of empirical studies to gain understanding into personal explanations of psychosis may not have been representative of the perspectives within public discourse. The inclusion of grey literature (eg, first person lived experience accounts of narrative that are publicly available and narratives which are conveyed in mediums in addition to text) may have greater nuances in personal explanations and reduces the likelihood of editing to suit a scientific style of writing.

Fifth, the consideration of only empirical studies can generate questions surrounding injustice. Empirical/scientific research can privilege voices of those who represent WEIRD countries and given the challenges experienced by academics with lived experience,^64,65^ that is academics who outwardly acknowledge their experiences of mental health problems within their research. There is increasingly literature supporting the inclusion of lived experience voices within academic spaces, yet university culture and systems may preclude or substantially make difficult the involvement of lived experience perspectives. To reduce these injustices that may be perpetuated by academic research, the team consisted of individuals with lived experience and the Lived Experience Advisory Panel was consulted throughout the development and conduct of the paper.

Last, the discussed limitations may have been perpetuated through the choice of information sources used in the search strategy. Whilst one social sciences/humanities electronic database (Scopus) was used in the search, a greater number of electronic databases with a biological or psychological focus (e.g., Medline, Embase, and PsycINFO) were used. Given the identification of mental health models across 5 domains (including consumer, social, and cultural perspectives) this may have skewed the papers included in the review.^1^

## Conclusion

Individuals with experiences of psychosis frequently form one or more personal explanations that account for their experiences. Narrating this explanation can help to provide a degree of comprehension to a previously incomprehensible experience. The explanations they utilized were grouped into themes: Physical and psychiatric explanations; Traumatic and adversarial explanations; Emotional explanations; Religious, spiritual, and magical explanations; No explanation. Multiple explanations were often held in tandem and worked to embed the experiences in the individuals personal and cultural context. The explanations should be considered with sensitivity within healthcare contexts, with implications for assessment, treatment, intervention, and outcome measures.

## Supplementary material

Supplementary material is available at Schizophrenia Bulletin Open online.

sgaf006_suppl_Supplementary_Materials_S1

sgaf006_suppl_Supplementary_Materials_S2

sgaf006_suppl_Supplementary_Materials_S3

sgaf006_suppl_Supplementary_Materials_S4

sgaf006_suppl_Supplementary_Materials_S5
