# Supplementary material for: Personal Explanations for Psychosis: A Systematic Review and Thematic Synthesis
Source: Schizophr Bull Open. 2025 Mar 4;6(1):sgaf006. doi: 10.1093/schizbullopen/sgaf006 (PMC12062962; doi:10.1093/schizbullopen/sgaf006)
Supplement: sgaf006_suppl_Supplementary_Materials_S1 [file sgaf006_suppl_supplementary_materials_s1.docx]

| **Set** | **Search Statement** |
| --- | --- |
|  | exp Psychotic disorders/ |
|  | (psychosis or psychoses or psychotic*).ti,ab. |
|  | Schizophrenia/ |
|  | (schizophrenic* or schizophrenia).ti,ab. |
|  | (delusion* or hallucinat* or mad or madness or insane or insanity or hearing voice*).ti,ab. |
|  | (Spiritual adj2 (emergenc* or awakening or crisis or crises)).ti,ab. |
|  | (Psychosis or psychotic or psychoses or psychotic disorder or hallucination or delusion or schizophrenia or schizophrenic or schizoaffective or brief psychotic disorder or delusional disorder or schizophreniform or first episode psychosis or late onset psychosis or hearing voices).mp. |
|  | (Spiritual awakening or spiritual emergency or spiritual crisis or altered state or consensus reality or non-consensus reality or mad or madness or non-ordinary state or non-ordinary experience or religious ecstasy or mystical experience or mystical psychosis or spiritual hallucination or religious hallucination or psychotic enlightenment or Spontaneous Spiritual Awakening or highly sensitive or unusual experience).mp. |
|  | 1 or 2 or 3 or 4 or 5 or 6 or 7 or 8 |
|  | (making meaning* or meaning making or giving meaning).ti,ab. |
|  | framework of meaning*.ti,ab. |
|  | (meaning adj2 personal).ti,ab. |
|  | (making sense or sense making or "meaning in life").ti,ab. |
|  | (explain* or subjective or understand* or causal or attribute* or cause or purpose).ti,ab. |
|  | (personal meaning or subjective meaning or individual meaning).ti,ab. |
|  | (explanatory model or cause or causal or attribution or trigger or purpose or reason).ti,ab. |
|  | (understanding or insight or personal construct or belief or explanation).ti,ab. |
|  | 10 or 11 or 12 or 13 or 14 or 15 or 16 or 17 |
|  | (lived experience* or life experience*).ti,ab. |
|  | ((personal or individual) and (account*1 or story or stories or perspective* or testimon* or view*1 or narrative* or explanation* or understand* or insight* or experience*)).ti,ab. |
|  | 19 or 20 |
|  | 9 and 18 and 21 |
|  | limit 22 to "qualitative (best balance of sensitivity and specificity)" |
|  | limit 23 to English language |
