# Supplementary material for: Personal Explanations for Psychosis: A Systematic Review and Thematic Synthesis
Source: Schizophr Bull Open. 2025 Mar 4;6(1):sgaf006. doi: 10.1093/schizbullopen/sgaf006 (PMC12062962; doi:10.1093/schizbullopen/sgaf006)
Supplement: sgaf006_suppl_Supplementary_Materials_S2 [file sgaf006_suppl_supplementary_materials_s2.docx]

| **Criteria** | **Include if:** | **Exclude if:** |
| --- | --- | --- |
| Criteria 1:  Publication Type | Paper is empirical research | Paper is not empirical research  Including:   - Dissertation thesis - Review (systematic, literature, book) - Summaries - Commentary - Book Chapter - Conference Abstract - Theory Paper - Opinion Piece - Protocol - Non-empirical Personal Account |
| Criteria 2:  Study Design | Paper utilises qualitative prose data collection methods  Including:   - Interview   - Semi-structured   - Structured, e.g. SEMI - Open ended survey question - Mixed methods where qualitative data is extractable | Paper does not utilise qualitative prose data collection methods  Including:   - Quantitative data - Case Report - Q Methodology - Qualitative methods which are not prose; poetry, photography |
| Criteria 3:  Population | Participant sample is non-organic psychosis or psychosis-like experience in adults   - Sample is >18 years: - Young adults - Adults - Older adults - Psychosis or psychosis-like experiences include: - Psychosis - Schizophrenia spectrum disorders - Attenuated Psychosis Syndrome - Psychosis-like experience - Anomalous self-experiences - At-risk mental state - Clinical high risk for psychosis - Ultra-high risk for psychosis - Prodrome - Hallucination - Delusion - Hearing voices - Spiritual emergency / crisis - Experiences of above in cultures which do not use the term psychosis | Participant sample is not non-organic psychosis or psychosis-like experience in adults   - Not non-organic psychosis, including: - Psychosis symptoms due to health condition e.g. Alzheimer’s, Epilepsy, delirium - Hallucinations due to sight loss e.g. Charles Bonnet Syndrome - Korsakoff's syndrome - Near death experience - Deathbed phenomena - Drug induced psychosis - SMI where psychosis is not specified - Sample is <18 years: - Youth (15-24) - Young people (15-24) - Adolescences (10-19) - Sample does not experience psychosis or PLE: - Carers - Clinicians - Family members |
| Criteria 4:  Outcome | Outcome regarding personal explanations of experiences | Outcome not regarding personal explanations of experiences |
