# Supplementary material for: Personal Explanations for Psychosis: A Systematic Review and Thematic Synthesis
Source: Schizophr Bull Open. 2025 Mar 4;6(1):sgaf006. doi: 10.1093/schizbullopen/sgaf006 (PMC12062962; doi:10.1093/schizbullopen/sgaf006)
Supplement: sgaf006_suppl_Supplementary_Materials_S4 [file sgaf006_suppl_supplementary_materials_s4.docx]

Supplementary Material 4: Full Codebook

| Theme | Subthemes | Includes | Illustrative Quote | Papers |
| --- | --- | --- | --- | --- |
| ***Medical model explanations*** | | | | 19 |
|  | Biological Causes or Injury | |  | 13 |
|  |  | Brain dysfunction  Chemical imbalance  Injury  Physical health conditions e.g. epilepsy, poor eyesight, heart condition  Surgery  Traumatic brain injury  Viral infection | “I believe that they come from inside my brain… Some brain dysfunction.” (P#002)  “My mental health challenges were triggered by having radical surgery. I mean it is - it is important to say this, so, before [the surgery] I hadn't experienced [mental health challenges].” (P#004)  “I've been told by my GP actually that, er with me having chickenpox as a child as well the virus can lay dormant which to me, erm, as well as religious beliefs it also, I believed very strongly they were proper visions but also having that virus lay dormant in me that, er, there is a medical side to it as well which to me is now, at long last been recognised…” (P#007) | P#003  P#004  P#007  P#008  P#010  P#011  P#012  P#014  P#015  P#020  P#022  P#023  P#024 |
|  | Genetics |  |  | 8 |
|  |  | Genetic causes  Genetic inheritance | “That's why it all happened because my mother was ill and I'm also ill. She was ill psychiatrically, and I'm also psychiatric. She has been in a closed psychiatric institute. I met about 30 doctors in my life from all sorts of hospitals. The doctor that admitted me the first time I was hospitalised already knew my mother; maybe her genes are in me...” (P#009)  “But I think there’s definitely something there genetically. It runs in the family because [my mum’s] mum was diagnosed … paranoid psychosis.” (P#021)  “My genes predisposed me to get ill… You cannot do anything about that.” (P#022) | P#005  P#008  P#009  P#010  P#012  P#021  P#022  P#023 |
|  | Psychological Illness | |  | 7 |
|  |  | Fragmentation of the mind  Mental illness  Prior anxiety disorder  Prior depressive disorder  Psychotic disorder | “I feel like they all linked like the psychosis came along with a lot of depression, anxiety. I think it started off with depression because, em… I did actually go onto some tablets, Fluoxetine, I think it was called? And, em, I think it all stemmed from depression and anxiety.” (P#P#001)  “My mind that’s become fragmented.” (P#021) | P#001  P#005  P#007  P#012  P#020  P#021  P#025 |
| ***Drug related explanations*** | | | | 9 |
|  |  | Alcohol  Non-prescription drugs, including cannabis, narcotics, LSD, amphetamines, psilocybin, cocaine  Prescription drugs | “So I had an ice (crystal methamphetamine) addiction and I think out of that came a number of…psychotic experiences.” (P#005)  “Ermm, I think it [narcotics] was definitely a contributing factor but I don’t think it was the sole reason why I lost my marbles.” (P#008)  “I was taking drugs… It is because of the drugs… Everything… Tablets… Cannabis… Tranquilizers.” (P#012)  “Heavy drinking.” (P#020) | P#005  P#008  P#010  P#011  P#012  P#013  P#018  P#020  P#022 |
| ***Physical Stress explanations*** | | | | 4 |
|  |  | Hunger  Physical overwork  Sleep deprivation | “I wasn’t sleeping it must have been and starving myself must have brought it on.” (P#013)  “I just thought it's a lack of sleep and also things in my past which I haven't discussed coming up. That's the reason I had, so what the doctor said didn't really matter.” (P#017) | P#013  P#014  P#017  P#020 |
| ***Trauma and adversities as explanations*** | | |  | 16 |
|  | Chronic or complex trauma | |  | 10 |
|  |  | Abuse, including: childhood, domestic, emotional, physical, sexual  Attachment trauma  Bullying  Exile  Family separation  Imprisonment  Neglect  Political violence  Seeking refuge  Warfare | “The Somoza guards kidnapped my husband and they cut his throat, I was working when I knew it and got in shock; so they took me to the hospital, since then I started to take pills and I still do.” (P#003)  “I come from a fairly dysfunctional family… It did actually take me years and years to put together the idea… That the mental health problems sort of arose as a means for my brain to externalise what was going on inside… Sometimes they (hallucinations) were more supportive than the people around me were… Maybe my brain created an environment for me… To be in… I understood better… I had control of.” (P#004)  “Back then my husband’s nerves were constantly on edge and I and my children had to be afraid when he started yelling at us. I couldn’t say anything or express my own thoughts. It was like a continuous stalemate situation, where we couldn’t communicate. Finally, I collapsed. I couldn’t sleep and I started to feel that everybody wanted something bad for me.” (P#006)  “In elementary school I was bullied constantly. So much that I wanted to kill myself. I think my psychosis started then, though nobody realized it. I started to hear their voices and I often saw those figures in the forest near our house.” (P#006) | P#003  P#004  P#006  P#008  P#010  P#013  P#016  P#017  P#020  P#025 |
|  | Single event trauma | |  | 8 |
|  |  | Assault  Bereavement  Traumatic accident  Traumatic birth  Traumatic injury | “[Care coordinator] said that is a possibility (bereavement) and when you think about it, it is true.” (P#008)  “It has something to do with the bereavement I imagine alright… I didn’t take time to grieve, I just wanted to get the thing settled… It was only with the dying off of the others that I had this terrible coping.” (P#014) | P#002  P#003  P#006  P#008  P#011  P#014  P#023  P#025 |
|  | Unspecified trauma | |  | 7 |
|  |  |  | “My brain was trying to process all the things happening around me, and tried to externalise it in some form that I could perhaps deal with a bit better…” (P#004)  “[I] began to see more and more, the links to trauma.” (P#005)  “[Voices are] either from a traumatic episode where you’re just becoming your own best friend…I believe it all started off as a traumatic experience.” (P#005) | P#002  P#004  P#005  P#008  P#017  P#020  P#022 |
|  | Adversity (transient or structural) | |  | 4 |
|  |  | Debt  Discrimination  Homelessness  Poverty  Social Exclusion  Societal Structures | “People should have money and should be given jobs because if you do not have money, you become stress[ed]. This stress exacerbates your illness. When you have money you don’t become mentally ill.” (P#011)  “I am strictly against monarchy… It is not good that a monarch not only rules but also takes possession of the people… This brought me down.” (P#022) | P#003  P#011  P#014  P#022 |
| ***Emotional explanations*** | | |  | 20 |
|  | Emotional experiences | |  | 15 |
|  |  | Stress, including: education-related, financial stress, general stress, work-related  Difficulty with emotional insight  Difficulty with nerves  Emotional suppression  Sensitive nature  Vulnerable to distress | “Prior my collapse, I worked so hard and I was sure that I’d get that promotion. However, they decided to hire another guy for that position, going right past me. It was horrible. I felt that they had mistreated me, and I started to think it was all just some kind of strange game.” (P#006)  “I worked for the post office for many years and I was good at it... and then I got fired. Since then, I haven't been able to find another job... I got depressed, so I decided to go to Israel... and here, in Israel, I became really ill with schizophrenia.” (P#009)  “I always thought I was not being good enough at work. My boss never said a word, but there always was this tense atmosphere.” (P#022) | P#001  P#005  P#006  P#008  P#009  P#010  P#014  P#015  P#017  P#018  P#019  P#020  P#022  P#023  P#025 |
|  | Interpersonal difficulties | |  | 15 |
|  |  | Family conflict  Loneliness  Relationship breakdowns  Relationship disappointments  Social altercations  Social anxieties  Social isolation  Social regrets  Social rejection  Social transgressions | “There were constant disappointments in our relationship and then that divorce thing happened. It affected my self-confidence, leading to other failures and disappointments. It was as if the house of cards that we had carefully built suddenly collapsed.” (P#006)  “So, my wife left me... And she took the kids, and I didn't see them... Not since they were 13... I saw them for half an hour and then they were gone... Difficult, very difficult. I loved them so much... That is why I became sick...” (P#009)  "I felt very isolated. I learnt for my exams, and some days I did not see anyone. At university there were so many students, but I was somewhat too afraid to contact anyone…” (P#022) | P#001  P#002  P#006  P#009  P#010  P#011  P#012  P#014  P#015  P#018  P#019  P#020  P#022  P#023  P#024 |
|  | Transitional periods | |  | 3 |
|  |  | Transition to adulthood  Transition to married life  Transition to motherhood | “I heard the voices when I got married. Yeah, I was ill… After birth… After the birth.” (P#013)  “It was OK until, as I say, I went to senior school, and then things got difficult, and you don't really talk about problems and things like that cos nobody really understands about it.” (P#018) | P#006  P#013  P#018 |
| ***Spiritual, religious, and magical explanations*** | | |  | 22 |
|  | Religious entities and phenomena | |  | 14 |
|  |  | Demons  Devil  Gift from God  God’s plan / Allah’s will / fate  God’s punishment  Presence of God  Presence of Holy Spirit  Religious groups  Role of a higher power | “I think I was touched by the Holy Spirit, that felt good, like I was being good or being rewarded for being good, whether that’s to do with schizophrenia I don’t know, it’s a good feeling, it is positive.” (P#008)  “Of course this is because of God. I mean, God gives you these problems but he also gives remedy. I now have these problems but there are also hospitals. I know that I have these problems as a result of stress, depression, accumulation, but I also know that they will make be feel better.” (P#016)  “I think that, erm, I was being guided by God throughout this time.” (P#017)  “We still believe that this is because of fate and previous deeds. We usually go to the temple and pray.” (P#023) | P#003  P#004  P#005  P#007  P#008  P#009  P#013  P#014  P#016  P#017  P#019  P#021  P#023  P#024 |
|  | Spiritual entities and phenomena | |  | 14 |
|  |  | Amafufunyana^[[1]](#footnote-1)^  Ancestors  Communication with supernatural world  Connection to universe  Djinn / Jinn / Jinnat^[[2]](#footnote-2)^  Evil forces / Dark entities  Karma / Punishment  Poltergeist  Souls  Spirits / Spiritual connection / Spiritual energies / Spiritual embodiment / Spiritual possession  Supernatural gift / Telepathy | “And I've told him about the experience with Jinns. Sometimes he was there! When I, like when the Jinns were there and he'd like feel it as well.” (P#001)  “A large part of me, because I have, still have got my own questions about chemicals in your body getting out of hand and what I believe in, erm, but yeah, a large of me does think that it was an evil spirit that, that's why I, that's why my body broke down.” (P#007)  “I never believed in anything, not in God, not in the “karma”, and then they told me I have schizophrenia; this is how the universe talks to me.” (P#009)  “It was amafufunyana caused by the ancestors.” (P#019) | P#001  P#002  P#004  P#007  P#013  P#016  P#018  P#019  P#020  P#021  P#022  P#023  P#024  P#025 |
|  | Magical entities and phenomena | |  | 11 |
|  |  | Magic based explanations, including:  Amafufunyana, Black magic, Curses, Jadoo^[[3]](#footnote-3)^, Nazar^[[4]](#footnote-4)^/Evil Eye, Sorcery, Taweez^[[5]](#footnote-5)^, Toor^[[6]](#footnote-6)^, Witchcraft | “I thought like, I thought it might be evil eye and I was thought I was stressed at the same time. So, both things stress and evil eye.” (P#001)  “There are bad people and witchcraft do exist and that was what happened to me. I did not believe in witchcraft before that, but now I do.” (P#003)  “I think I've been hexed... I think that's why I'm sick... They put something in my coffee... The Rabbi from Jaffa gave me an amulet. It lasted for two and a half years... then its power wore off. The Indian woman, she removed my hex... removed my evil eye... It would last for two months, but then it came back.” (P#009)  “No, well, I thought [it was witchcraft] because the people who, there were some people I didn’t get along with… But I felt it like it was the cause of being exposed to someone, without knowing who, that I had eaten something or they had given me something or something like that.” (P#010) | P#001  P#003  P#009  P#010  P#011  P#014  P#019  P#020  P#022  P#023  P#024 |
| ***No explanation*** | | |  | 9 |
|  | Explanation not known | |  | 6 |
|  |  |  | “I dunno. It's just, there's something unexplainable, to be honest.” (P#001) | P#001  P#019  P#020  P#022  P#023  P#024 |
|  | Explanation not sought | |  | 2 |
|  |  |  | “I have not really put thought into it because it’ll just mess my head up if I put thought into it… I don’t want to mess around with something like because it’s just pointless thinking about it, there’s nothing it’s just fruitless.” (P#008)  “I don't talk to other people about it. I just want to forget about it now.” (P#017) | P#008  P#017 |
|  | Explanation not found | |  | 2 |
|  |  |  | “There has got to be a reason why, that’s what I wanted to find out, if you get a cut on your hand you can see it, you can see it getting worse, whereas something inside you can’t see. There is nothing obvious.” (P#008) | P#008  P#025 |

Table 2: Reference Key

| Key | Reference |
| --- | --- |
| P#001 | Patel, K., Cardno, A., & Isherwood, T. (2023). 'Like I said about culture. You don't talk about mental health': An interpretative phenomenological analysis of the experience of first-episode psychosis in South Asian individuals. Early Intervention in Psychiatry, 17(8), 771-783. https://doi.org/https://dx.doi.org/10.1111/eip.13368 |
| P#002 | Pigeon-Gagné, E., Vigu, T., Kadio, K., Bonnet, E., & Ridde, V. (2022). Explanatory models of psychotic-like experiences in rural Burkina Faso: A qualitative study among indigents and their community. SSM - Mental Health, 2, 100166. https://doi.org/https://dx.doi.org/10.1016/j.ssmmh.2022.100166 |
| P#003 | Lyons, M., Evison, P., Berrios, R., Castro, S., & Brooks, H. (2022). The lived experience of psychosis in Nicaragua: a qualitative examination of the views of service users. Journal of Mental Health, 31(1), 50-57. https://doi.org/https://dx.doi.org/10.1080/09638237.2020.1844871 |
| P#004 | McGranahan, R., Jakaite, Z., Edwards, A., Rennick-Egglestone, S., Slade, M., & Priebe, S. (2021). Living with Psychosis without Mental Health Services: A Narrative Interview Study. BMJ Open, 11(7), e045661. https://doi.org/10.1136/bmjopen-2020-045661 |
| P#005 | Clements, S., Coniglio, F., & Mackenzie, L. (2020). “I’m Not Telling an Illness Story. I’m Telling a Story of Opportunity”: Making Sense of Voice Hearing Experiences [Article]. Community Mental Health Journal, 56(2), 196-205. https://doi.org/10.1007/s10597-019-00465-x |
| P#006 | Bergström, T., Seikkula, J., Holma, J., Mäki, P., Köngäs-Saviaro, P., & Alakare, B. (2019). How do people talk decades later about their crisis that we call psychosis? A qualitative study of the personal meaning-making process [Article]. Psychosis, 11(2), 105-115. https://doi.org/10.1080/17522439.2019.1603320 |
| P#007 | Marriott, M., Thompson, A., Cockshutt, G., & Rowse, G. (2019). Narrative insight in psychosis: The relationship with spiritual and religious explanatory frameworks. Psychology and Psychotherapy: Theory, Research and Practice, 92(1), 74-90. https://doi.org/10.1111/papt.12178 |
| P#008 | Carter, L., Read, J., Pyle, M., & Morrison, A. (2018). "I Believe I Know Better Even than the Psychiatrists What Caused It": Exploring the Development of Causal Beliefs in People Experiencing Psychosis. Community Ment Health J, 54(6), 805-813. https://doi.org/10.1007/s10597-017-0219-3 |
| P#009 | Araten-Bergman, T., Avieli, H., Mushkin, P., & Band-Winterstein, T. (2016). How aging individuals with schizophrenia experience the self-etiology of their illness: a reflective lifeworld research approach [Article]. Aging and Mental Health, 20(11), 1147-1156. https://doi.org/10.1080/13607863.2015.1063110 |
| P#010 | Mora-Rios, J., Ortega-Ortega, M., & Natera, G. (2016). Subjective Experience and Resources for Coping With Stigma in People With a Diagnosis of Schizophrenia: An Intersectional Approach. Qualitative Health Research, 26(5), 697-711. https://doi.org/https://dx.doi.org/10.1177/1049732315570118 |
| P#011 | Brooke-Sumner, C., Lund, C., Petersen, I. . (2014). Perceptions of psychosocial disability amongst psychiatric service users and caregivers in South Africa. African Journal of Disability, 3(1). https://doi.org/https://doi.org/10.4102/ajod.v3i1.146 |
| P#012 | Fenekou, V., & Georgaca, E. (2010). Exploring the experience of hearing voices: A qualitative study [Article]. Psychosis, 2(2), 134-143. https://doi.org/10.1080/17522430903191783 |
| P#013 | Cookson, A., & Dickson, J. M. (2010). The subjective experiences of people with an intellectual disability and diagnosis of schizophrenia who are detained in a medium secure unit [Article]. Journal of Applied Research in Intellectual Disabilities, 23(4), 379-389. https://doi.org/10.1111/j.1468-3148.2010.00554.x |
| P#014 | Quin, R. C., Clare, L., Ryan, P., & Jackson, M. (2009). 'Not of this world': The subjective experience of late-onset psychosis. Aging and Mental Health, 13(6), 779-787. https://doi.org/https://dx.doi.org/10.1080/13607860903046453 |
| P#015 | Judge, A., Estroff, S., Perkins, D., & Penn, D. (2008). Recognizing and Responding to Early Psychosis: A Qualitative Analysis of Individual Narratives. Psychiatric Services, 59(1), 96-99. https://doi.org/10.1176/ps.2008.59.1.96 |
| P#016 | Leavey, G., Guvenir, T., Haase-Casanovas, S., & Dein, S. (2007). Finding help: Turkish-speaking refugees and migrants with a history of psychosis. Transcult Psychiatry, 44(2), 258-274. https://doi.org/10.1177/1363461507077725 |
| P#017 | Perry, B. M., Taylor, D., & Shaw, S. K. (2007). "You've got to have a positive state of mind": An interpretative phenomenological analysis of hope and first episode psychosis [Article]. Journal of Mental Health, 16(6), 781-793. https://doi.org/10.1080/09638230701496360 |
| P#018 | Hirschfeld, R., Smith, J., Trower, P., & Griffin, C. (2005). What do psychotic experiences mean for young men? A qualitative investigation. Psychology and Psychotherapy: Theory, Research and Practice, 78(2), 249-270. https://doi.org/https://doi.org/10.1348/147608305X25865 |
| P#019 | Lund, C., & Swartz, L. (1998). Xhosa-Speaking Schizophrenic Patients' Experience of Their Condition: Psychosis and Amafufunyana. South African Journal of Psychology, 28(2), 62-70. https://doi.org/10.1177/008124639802800202 |
| P#020 | McCabe, R., & Priebe, S. (2004). Explanatory models of illness in schizophrenia: comparison of four ethnic groups. Br J Psychiatry, 185, 25-30. https://doi.org/10.1192/bjp.185.1.25 |
| P#021 | Lewis, S. H., Sanderson, C., Gupta, A., & Klein, C. (2020). “Maybe it’s kind of normal to hear voices”: The role of spirituality in making sense of voice hearing. Journal of Spirituality in Mental Health, 22(1), 49-64. https://doi.org/10.1080/19349637.2018.1520183 |
| P#022 | Conrad, R., Schilling, G., Najjar, D., Geiser, F., Sharif, M., & Liedtke, R. (2007). Cross-cultural comparison of explanatory models of illness in schizophrenic patients in Jordan and Germany. Psychol Rep, 101(2), 531-546. https://doi.org/10.2466/pr0.101.2.531-546 |
| P#023 | Saravanan, B., Jacob, K. S., Johnson, S., Prince, M., Bhugra, D., & David, A. S. (2007). Belief models in first episode schizophrenia in South India. Soc Psychiatry Psychiatr Epidemiol, 42(6), 446-451. https://doi.org/10.1007/s00127-007-0186-z |
| P#024 | Awan, N. R., Zahoor, N., Irfan, M., Naeem, F., Nazar, Z., Farooq, S., & Jahangir, F. (2015). BELIEFS ABOUT ILLNESS OF PATIENTS WITH SCHIZOPHRENIA. Journal of Postgraduate Medical Institute, 29(2). https://jpmi.org.pk/index.php/jpmi/article/view/1343 |
| P#025 | Holt, L., & Tickle, A. (2015). "Opening the curtains": How do voice hearers make sense of their voices? [Article]. Psychiatric Rehabilitation Journal, 38(3), 256-262. https://doi.org/10.1037/prj0000123 |

1. An illness experience of Xhosa people related to demonic possession, thought to be caused by sorcery [↑](#footnote-ref-1)
2. Supernatural creatures or spirits of good or evil intent [↑](#footnote-ref-2)
3. Jadoo or Jadu: Magic [↑](#footnote-ref-3)
4. The Evil Eye: a curse caused by the jealousy of another [↑](#footnote-ref-4)
5. Taweez, Tawiz, Ta’wiz: traditionally a protective amulet, used in this context to refer to black magic [↑](#footnote-ref-5)
6. Toor: Magic, bewitchment [↑](#footnote-ref-6)
