# Supplementary material for: Personal Explanations for Psychosis: A Systematic Review and Thematic Synthesis
Source: Schizophr Bull Open. 2025 Mar 4;6(1):sgaf006. doi: 10.1093/schizbullopen/sgaf006 (PMC12062962; doi:10.1093/schizbullopen/sgaf006)
Supplement: sgaf006_suppl_Supplementary_Materials_S5 [file sgaf006_suppl_supplementary_materials_s5.docx]

| **Paper** | **Country of Study** | **HAQ (WHO^1^)** |
| --- | --- | --- |
| 002 | Burkina Faso | 43 |
| 024 | Pakistan | 45 |
| 022* | Jordan | 60 |
| 023 | India | 61 |
| 011 | South Africa | 67 |
| 019 | South Africa | 67 |
| 003 | Nicaragua | 70 |
| 010 | Mexico | 74 |
| 012 | Greece | 78 |
| 006 | Finland | 83 |
| 014 | Ireland | 83 |
| 015 | USA | 83 |
| 009 | Israel | 84 |
| 022* | Germany | 86 |
| 005 | Australia | 87 |
| 001 | England | 88 |
| 004 | England | 88 |
| 007 | England | 88 |
| 008 | England | 88 |
| 013 | England | 88 |
| 016 | England | 88 |
| 017 | England | 88 |
| 018 | England | 88 |
| 020 | England | 88 |
| 021 | England | 88 |
| 025 | England | 88 |

1. Institute for Health Metrics and Evaluation, I., *HAQ Index (IHME (2017))*, O.W.i. Data., Editor. 2017.
